# Supplementary figures and images for: Caesarean section or vaginal delivery for low-risk pregnancy? Helping women make an informed choice in low- and middle-income countries
Source: PLOS Glob Public Health. 2022 Nov 14;2(11):e0001264. doi: 10.1371/journal.pgph.0001264 (PMC10022020; doi:10.1371/journal.pgph.0001264)

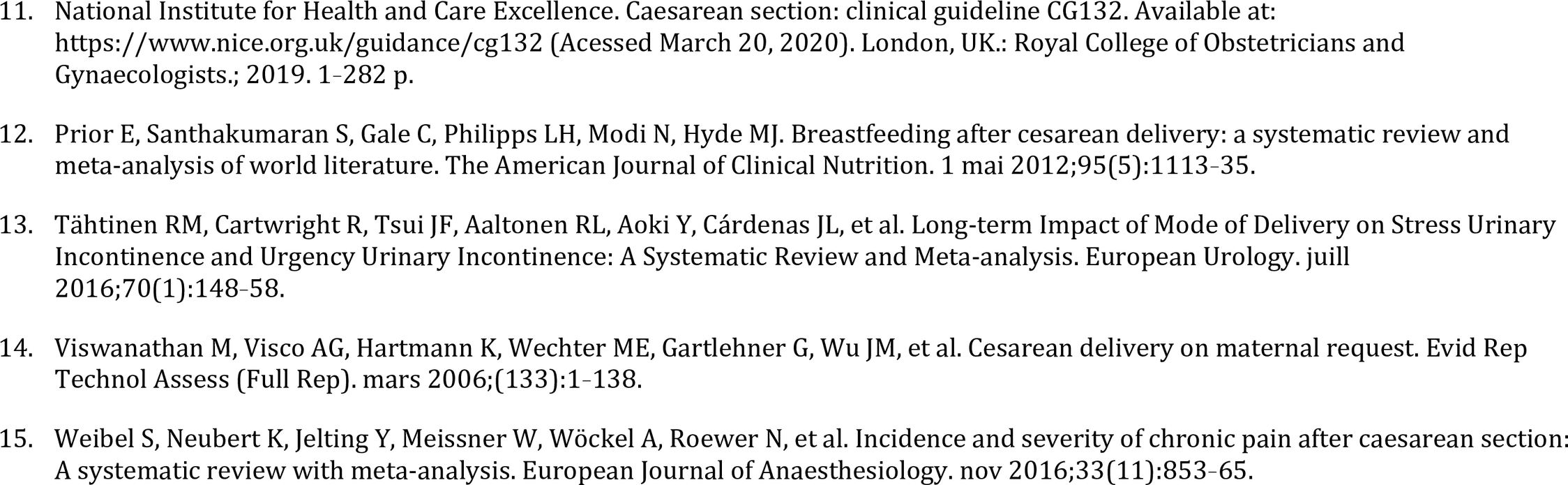

Supplement: S1 Table — (ZIP) [file pgph.0001264.s001.zip › S1_Table/S1_Table_5.tif]

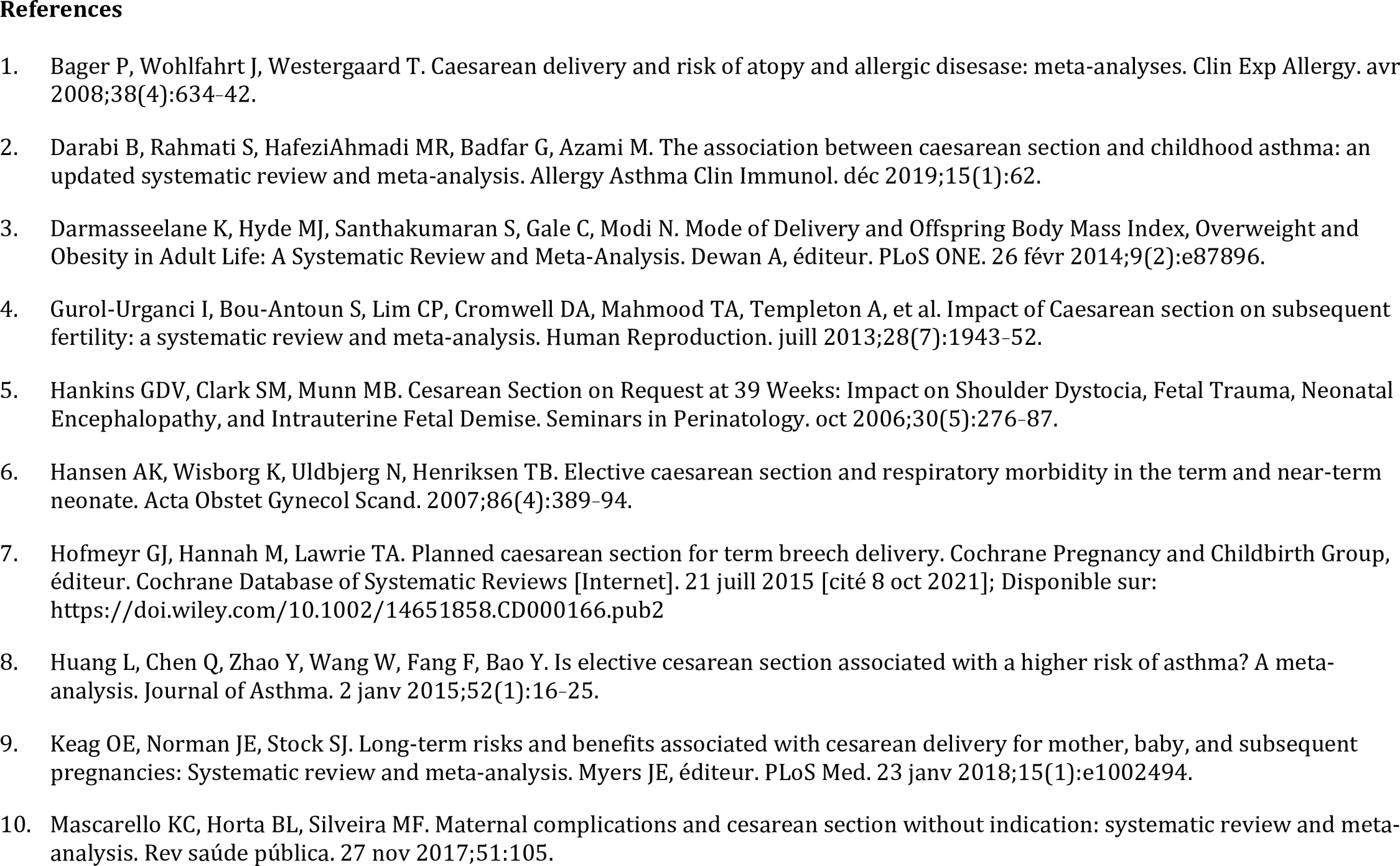

Supplement: S1 Table — (ZIP) [file pgph.0001264.s001.zip › S1_Table/S1_Table_4.tif]

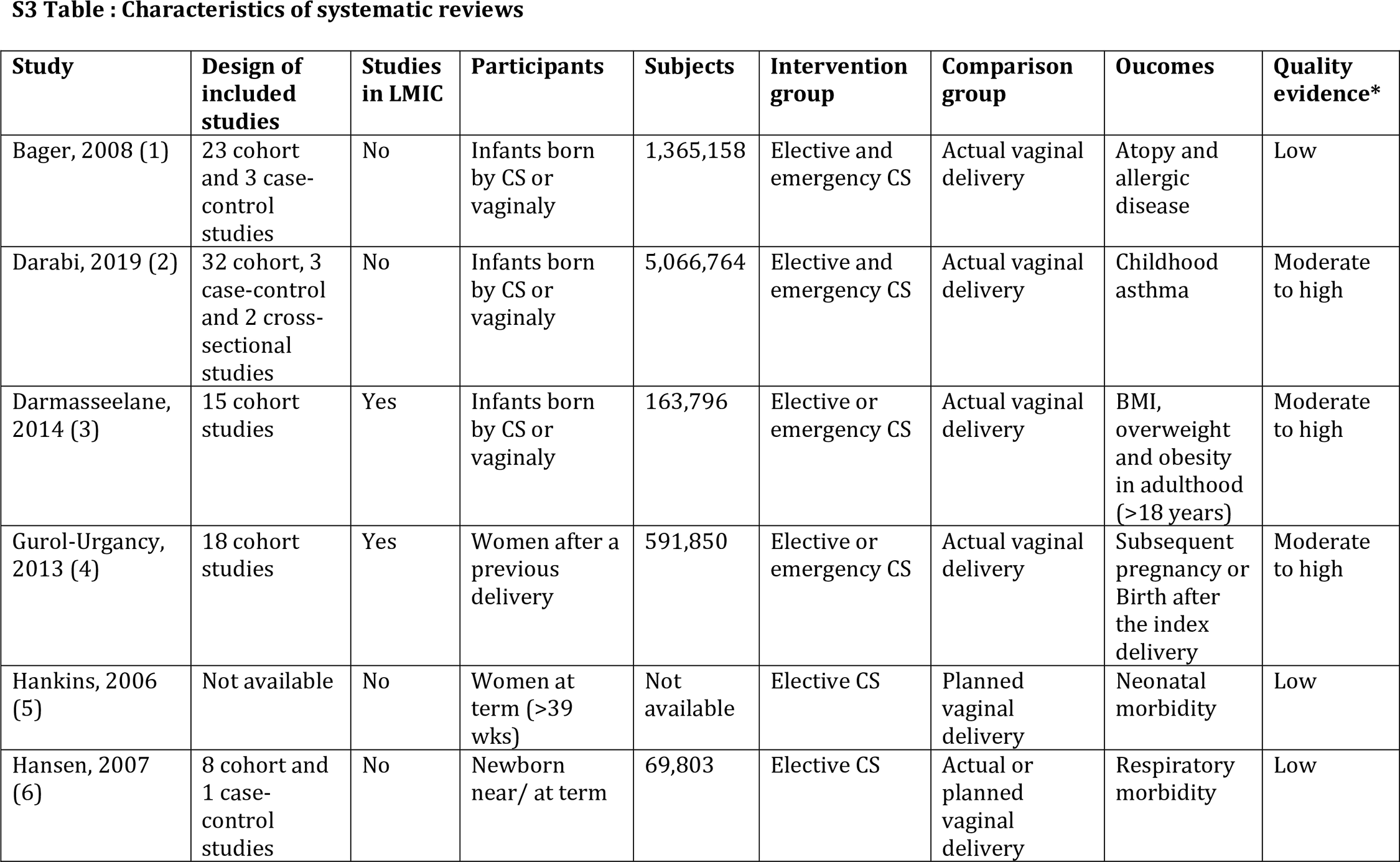

Supplement: S1 Table — (ZIP) [file pgph.0001264.s001.zip › S1_Table/S1_Table_1.tif]

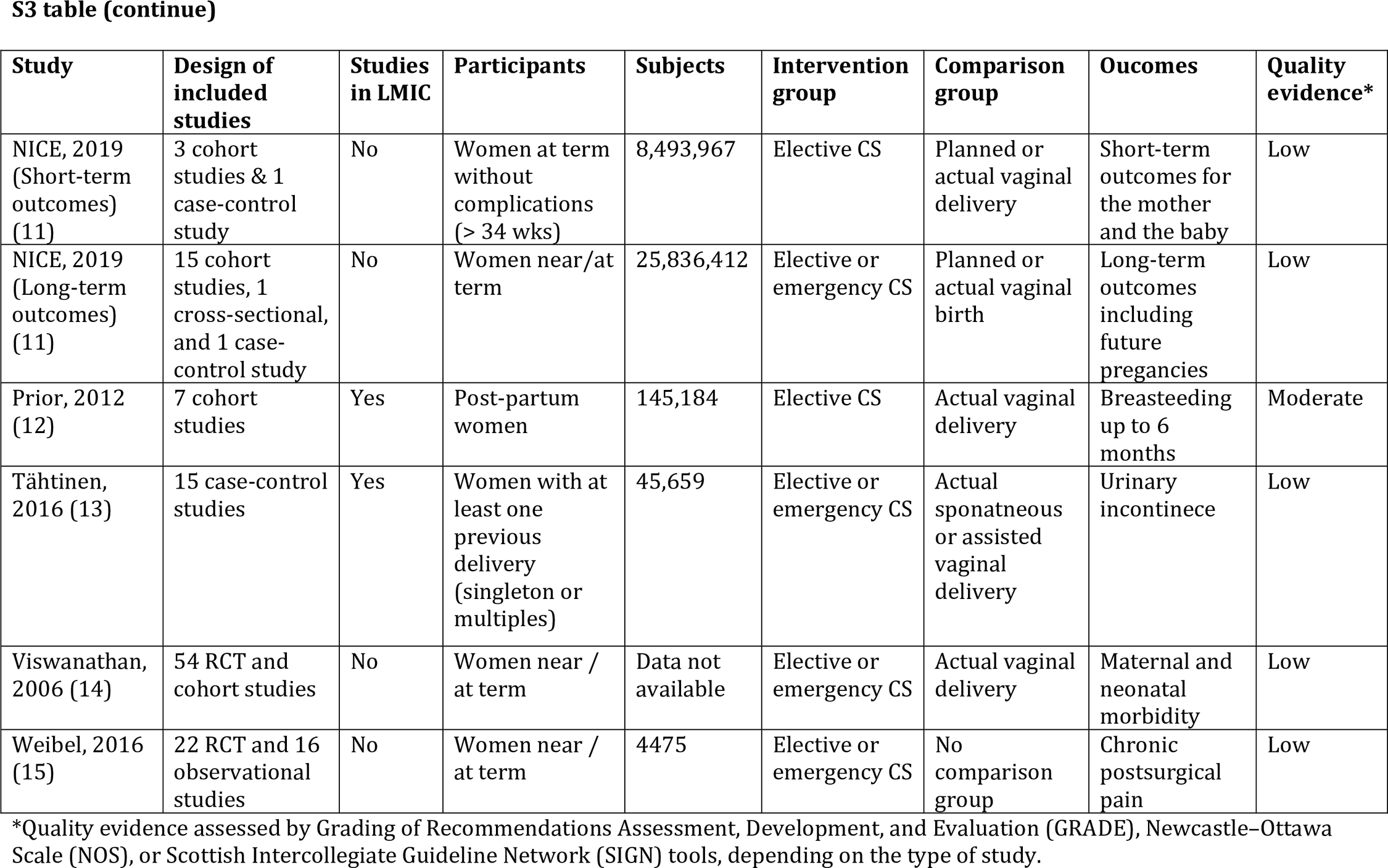

Supplement: S1 Table — (ZIP) [file pgph.0001264.s001.zip › S1_Table/S1_Table_3.tif]

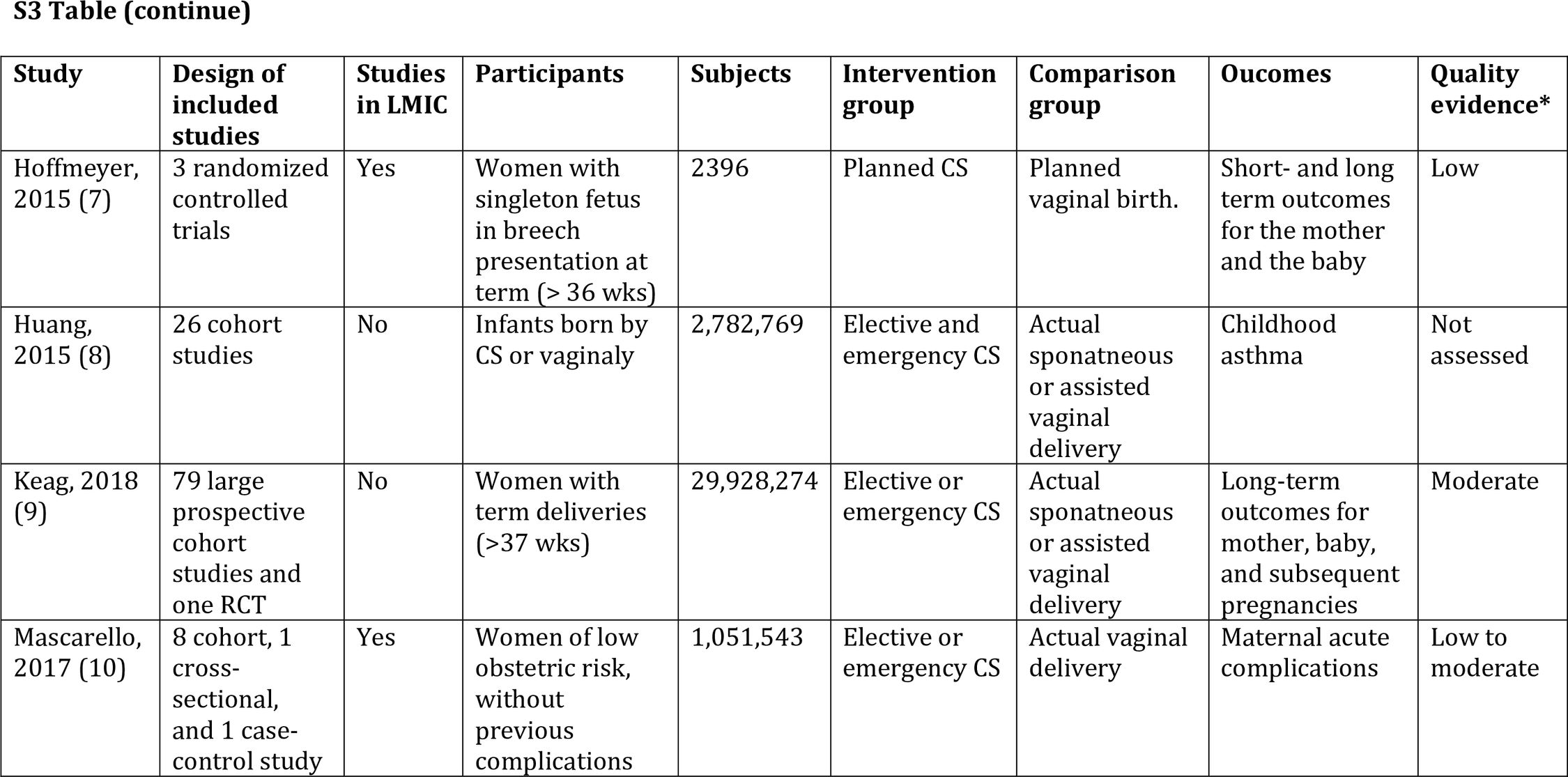

Supplement: S1 Table — (ZIP) [file pgph.0001264.s001.zip › S1_Table/S1_Table_2.tif]

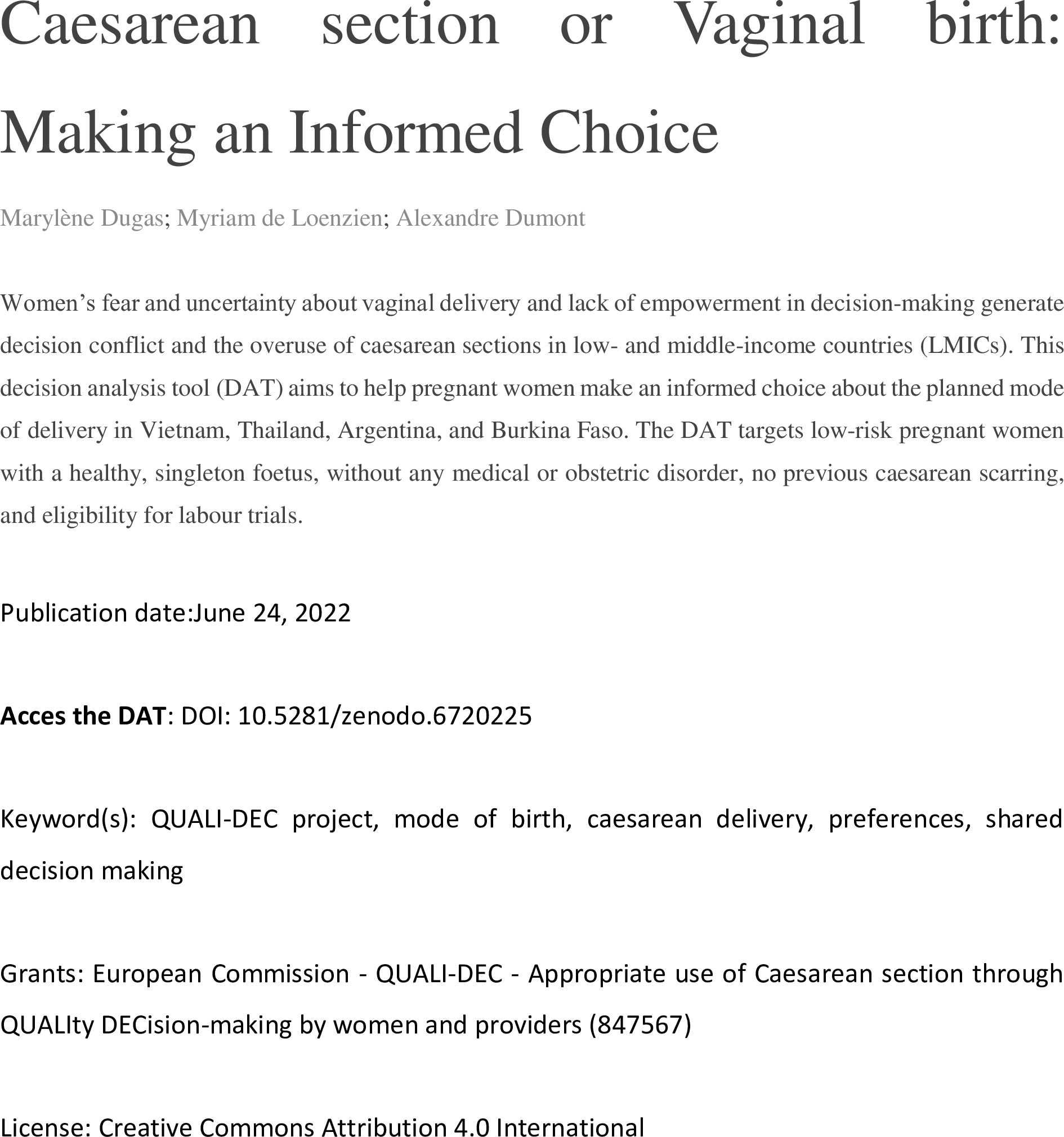

Supplement: S1 Text — (TIF) [file pgph.0001264.s002.tif]

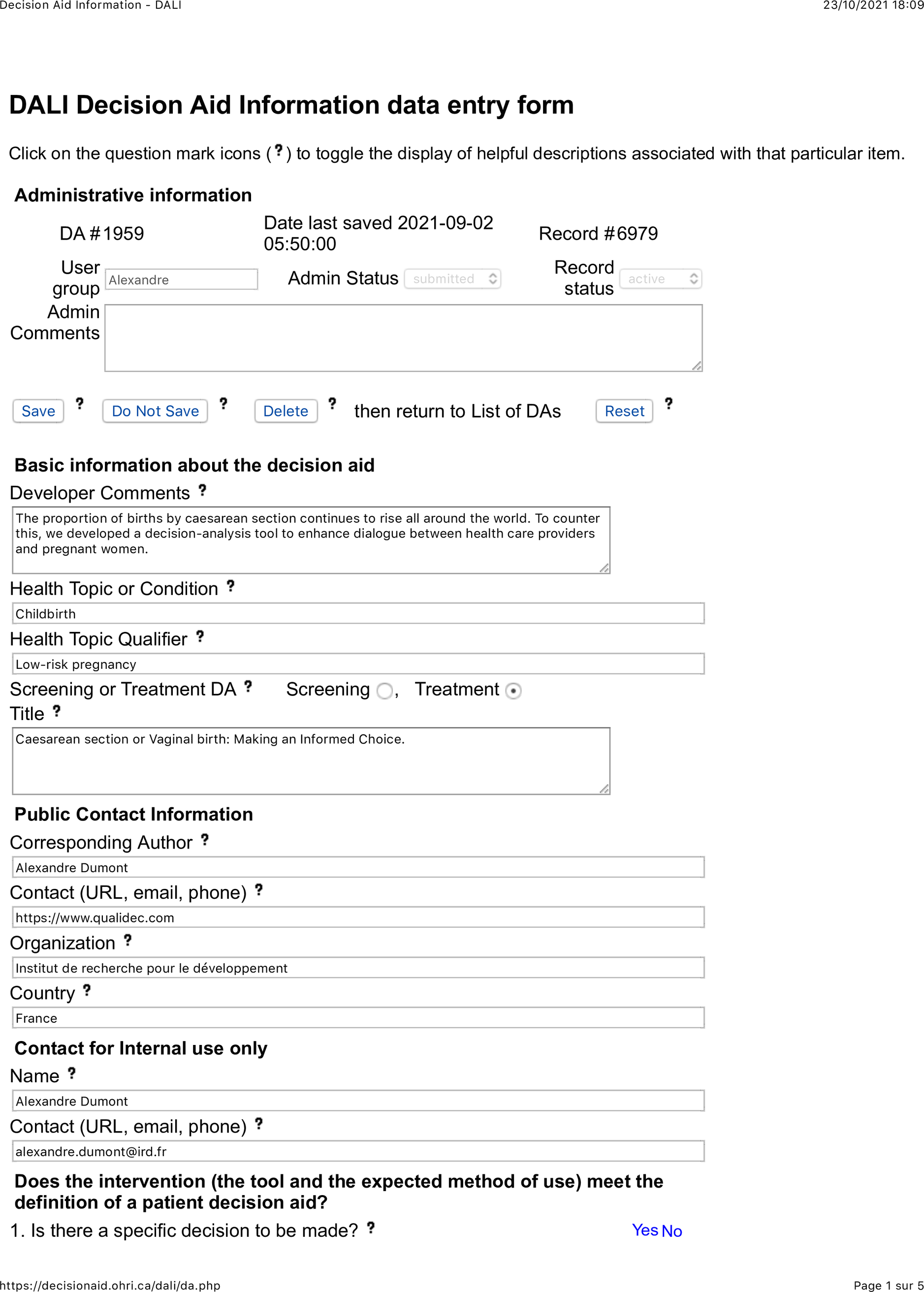

Supplement: S2 Text — (ZIP) [file pgph.0001264.s003.zip › S2_Text/S2_Text_1.tif]

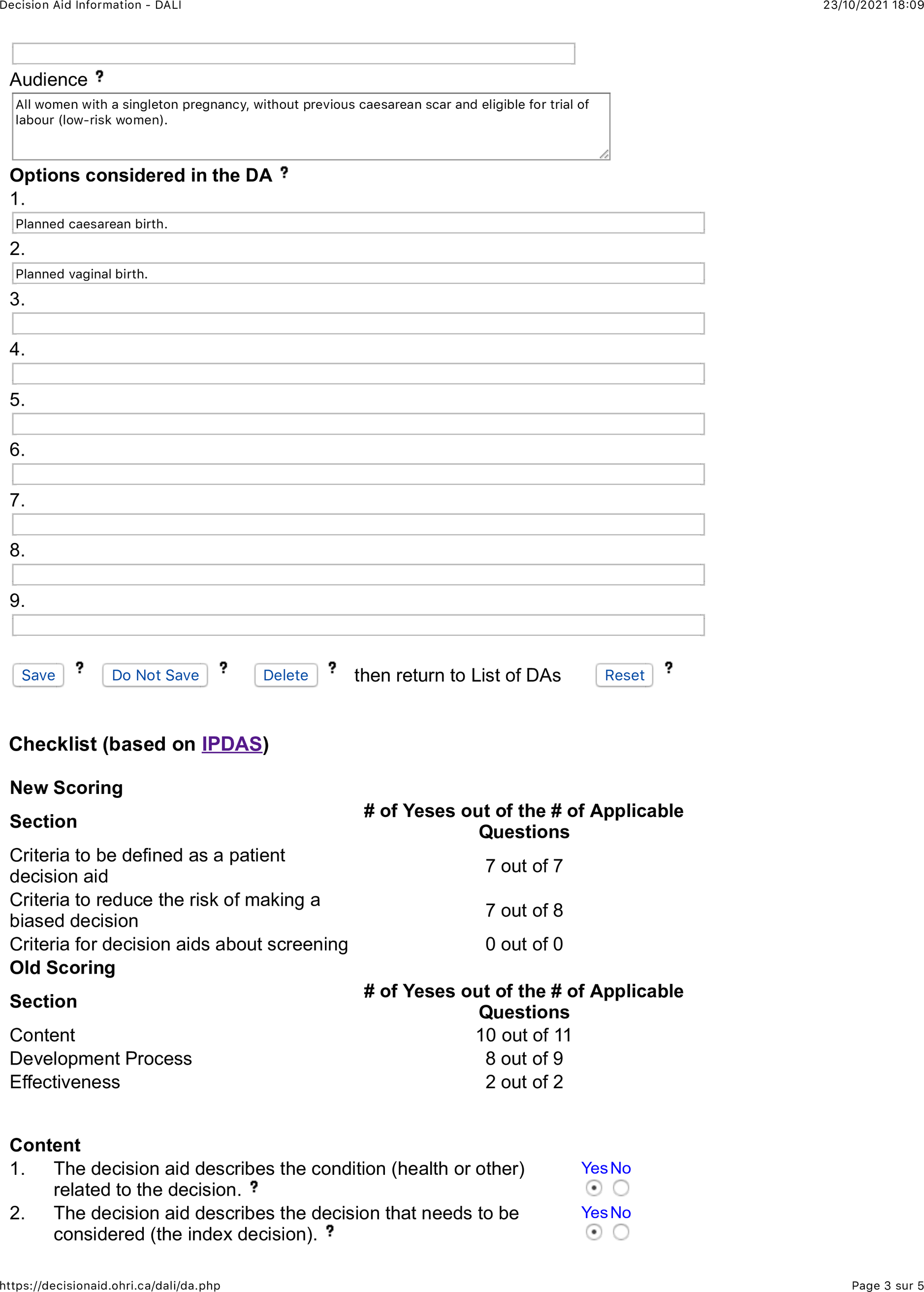

Supplement: S2 Text — (ZIP) [file pgph.0001264.s003.zip › S2_Text/S2_Text_3.tif]

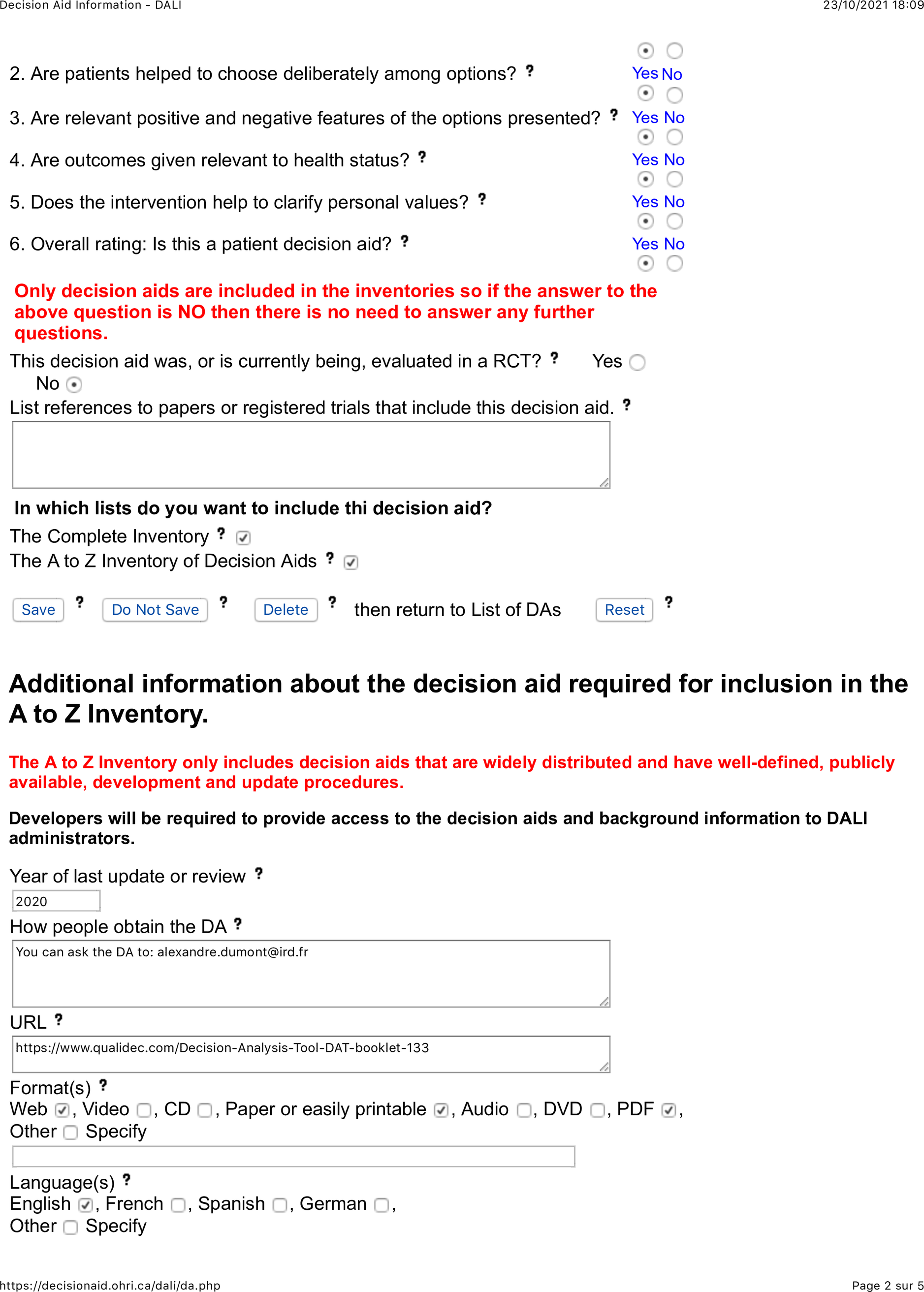

Supplement: S2 Text — (ZIP) [file pgph.0001264.s003.zip › S2_Text/S2_Text_2.tif]

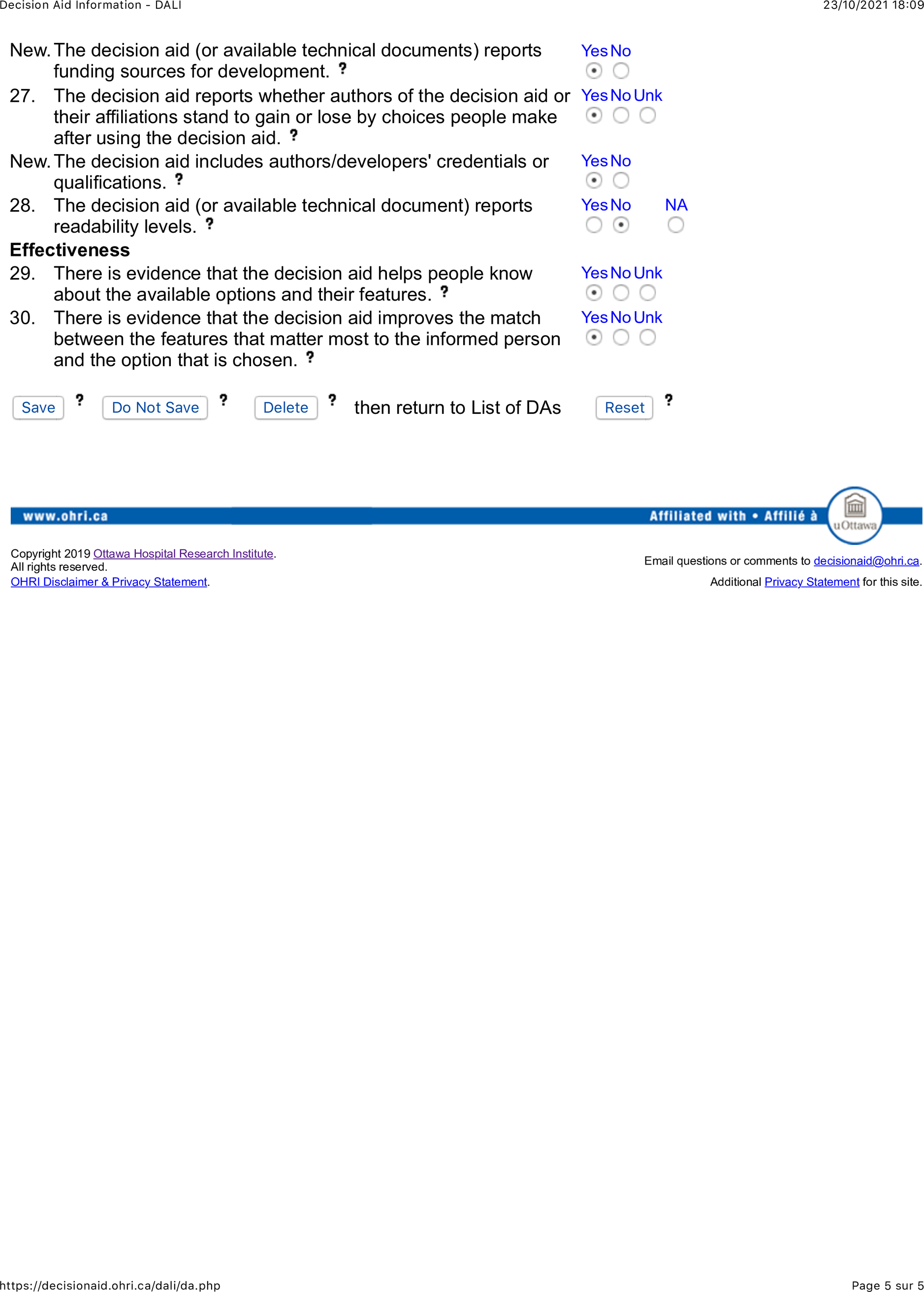

Supplement: S2 Text — (ZIP) [file pgph.0001264.s003.zip › S2_Text/S2_Text_5.tif]

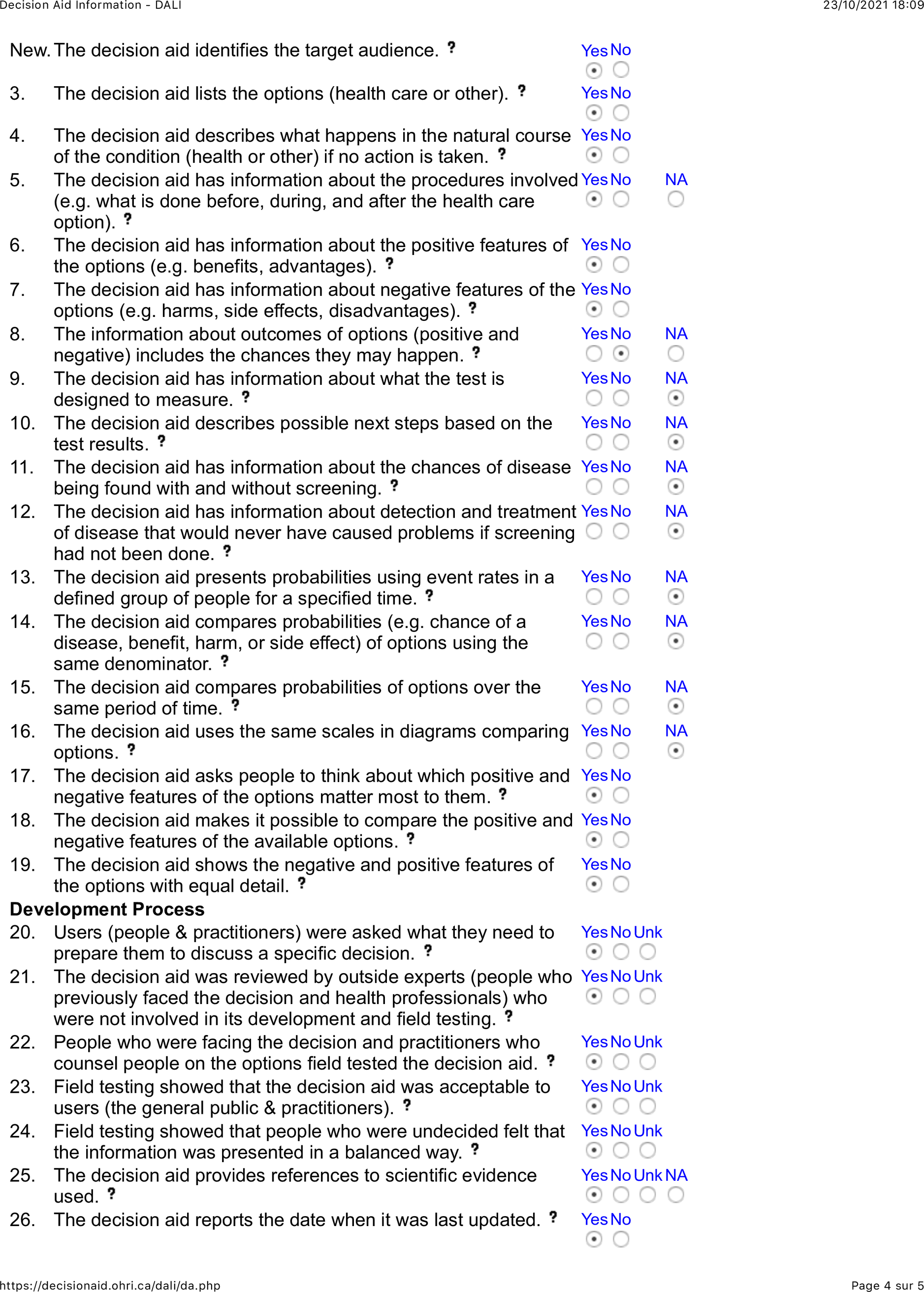

Supplement: S2 Text — (ZIP) [file pgph.0001264.s003.zip › S2_Text/S2_Text_4.tif]

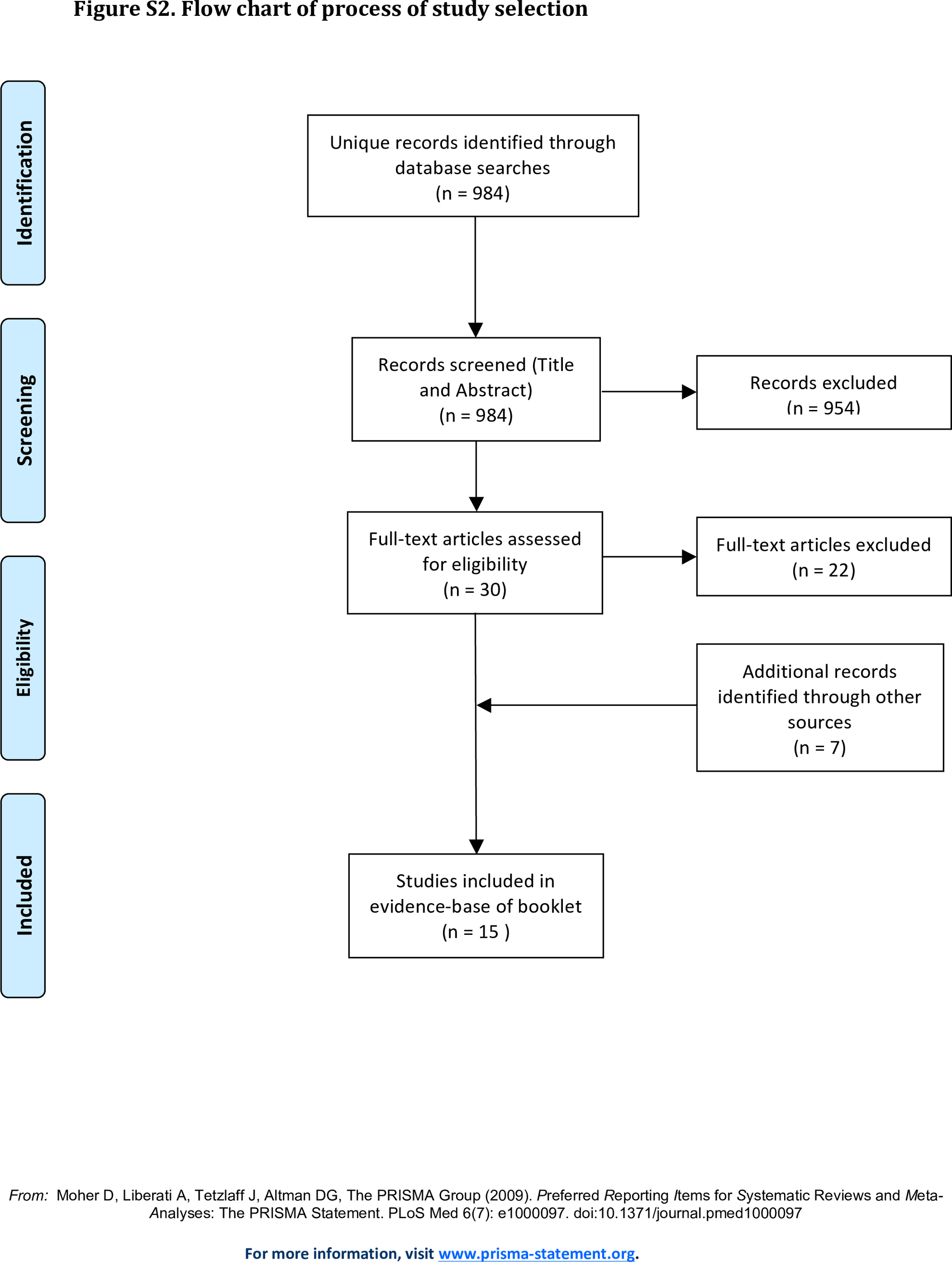

Supplement: S1 Fig — (TIF) [file pgph.0001264.s005.tif]
