# Supplementary material for: Caesarean section or vaginal delivery for low-risk pregnancy? Helping women make an informed choice in low- and middle-income countries
Source: PLOS Glob Public Health. 2022 Nov 14;2(11):e0001264. doi: 10.1371/journal.pgph.0001264 (PMC10022020; doi:10.1371/journal.pgph.0001264)
Supplement: Attachment — Submitted filename: Response to reviewers.docx [file pgph.0001264.s006.docx]

PGPH-D-21-01106
Caesarean section or vaginal delivery for low-risk pregnancy? Helping women make an informed choice in low- and middle-income countries
PLOS Global Public Health

**Answer to the editor and reviewers.**

Editor:

| Editor’s comment | Authors’ answer |
| --- | --- |
| 1. Please note that PLOS Global Public Health has specific guidelines on code sharing for submissions in which author-generated code underpins the findings in the manuscript. In these cases, all author-generated code must be made available without restrictions upon publication of the work. Please review our guidelines at <https://journals.plos.org/globalpublichealth/s/materials-and-software-sharing#loc-sharing-code> and ensure that your code is shared in a way that follows best practice and facilitates reproducibility and reuse. | Author-generated code is shared in a way that follows best practice and facilitates reproducibility and reuse |
| 2. Please include a complete copy of PLOS’ questionnaire on inclusivity in global research in your revised manuscript. Our policy for research in this area aims to improve transparency in the reporting of research performed outside of researchers’ own country or community. The policy applies to researchers who have travelled to a different country to conduct research, research with Indigenous populations or their lands, and research on cultural artefacts. The questionnaire can also be requested at the journal’s discretion for any other submissions, even if these conditions are not met.  Please find more information on the policy and a link to download a blank copy of the questionnaire here: <https://journals.plos.org/globalpublichealth/s/best-practices-in-research-reporting>. Please upload a completed version of your questionnaire as Supporting Information when you resubmit your manuscript. | We included PLOS’s questionnaire in supporting information |
| 3. Please amend your detailed online Financial Disclosure statement. This is published with the article. It must therefore be completed in full sentences and contain the exact wording you wish to be published. | We amended our detailed online Financial Disclosure statement |
| a. State the initials, alongside each funding source, of each author to receive each grant. b. State what role the funders took in the study. If the funders had no role in your study, please state: “The funders had no role in study design, data collection and analysis, decision to publish, or preparation of the manuscript.” | We add the sentence: “The funders had no role in study design, data collection and analysis, decision to publish, or preparation of the manuscript.” |
| 4. Please update your online Competing Interests statement. If you have no competing interests to declare, please state: “The authors have declared that no competing interests exist.” | We add the sentence: “The authors have declared that no competing interests exist.” |
| 5. We ask that a manuscript source file is provided at Revision. Please upload your manuscript file as a .doc, .docx, or .rtf. | We uploaded our manuscript file as .docx |
| 6. Please provide separate figure files in .tif or .eps format and remove any figures embedded in your manuscript file. Please also ensure that all files are under our size limit of 10MB. | We provided figure files in .tif format |
| For more information about how to convert your figure files please see our guidelines: <https://journals.plos.org/globalpublichealth/s/figures> |  |

Reviewer #1:

| Reviewer’s comment | Authors’ answer |
| --- | --- |
| This manuscript describes the formative research and process used to develop and assess a decision aid tool targeting improving informed decision-making around mode of delivery for low-risk women. This is an important area for research both because of the rising global cesarean delivery rates and the need to provide tools to improve communication and shared-decision-making models between pregnant/intrapartum women and their clinicians. | Thank you |
| Overall the manuscript is well presented and clear to understand. There are a few points of clarification and other suggestions for the authors. | Thank you for the relevant suggestions |
| Introduction: The authors can consider pointing out regional differences in preferences and rationale for CS or noting the lack of data from certain regions. For e.g. data from sub-Saharan Africa is quite limited, and that available does not suggest that many women are requesting CS in this region, thus stating that in general twice as many women from LMICs request CS compared to women in high-income countries may be an over generalization. | We changed the sentence page 3 to avoid over generalization. |
| Methods/Results : |  |
| • In both the pre-DAT development interviews noted in Step 1 and the interviews and focus group discussions conducted in Step 3, how were participants selected? The authors note which groups of participants were included but now how they were chosen for interviews. | We detailed recruitment and sampling of women and providers in the revised manuscript, page 6 and 9 |
| • For the interviews/focus group discussions held in Step 3, it is a bit unclear which version of the draft DAT participants were asked about - is it the version from January 2020 described in the results? If so it is a bit confusing to have the actually description of the DAT come after the results on the evaluation of the DAT. | Participants were asked about the first draft of the DAT based on the first overview of the literature in 2018. Changes were made page 8 to clarify this point. |
| • In Table 1: consider including some description/delineation of which interviews were pre-development of the DAT and which were performed after? | Table 2 was revised as suggested (page 11) |
| • How did the authors determine which of the summary of maternal/neonatal outcomes from the systematic review to include in the DAT. Was everything included or was it based on the GRADE scale result? If some were included in the DAT and not others, I would suggest noting this in Table 3. | All outcomes identified in the literature (with low or moderate certainty of evidence) were included in the DAT |
| • For the assessment of the DAT, did the interviews/ Focus groups address questions the optimal timing of when the DAT should be used i.e., when during antenatal care? | Yes, we asked the participants about the optimal timing of when the DAT should be used. Most women indicated that the last trimester is the appropriate time (page 17 of the revised manuscript). |
| • Given the DAT was developed for use in low-risk women without an indication for CS, is the overall premise of the DAT that generally vaginal birth is considered the route of choice or is it left open-ended with planned vaginal birth vs planned CS as equal contenders? This question speaks to the finding among some providers that perhaps this tool should be used more for women who request a planned CS. | The general premise of the DAT is to inform low-risk women about the risks and benefits of planned modes of birth and to ask them for their preference, without being biased toward one or the other option. We believe that this information can benefit all women, including those who prefer a planned vaginal birth. Of course, we hope that the DAT can also change the minds of women who initially say they prefer a cesarean section. |

Reviewer #2:

| Reviewer’s comment | Authors’ answer |
| --- | --- |
| Thank you for the opportunity to review the manuscript entitled, “Caesarean section or vaginal delivery for low-risk pregnancy? Helping women make an informed choice in low- and middle-income countries.” |  |
| SUMMARY: |  |
| The authors have presented the design, process, and evaluation of a decision analysis tool to support women/birthing people’s decision around birth type (vaginal or caesarean). Through a systematic review and qualitative approach in 4 countries, the authors have shared the experience of putting the tool together and its acceptability for implementation. The authors are to be commended for designing a tool to address an unmet need and providing an implementation pathway with both a paper tool and digital app. While I am enthusiastic about the paper and related findings, there are a few comments for authors’ consideration to improve the manuscript, particularly around streamlining and clarity of the messaging. | Thank you for your suggestions. |
| MAJOR: |  |
| • As part of the literature review for the evidence-based information, did the systematic review include international, national, or local guidelines and standards (i.e. World Health Organization guidelines, Ministry of Health guidelines in the 4 countries)? Can authors share more information on in inclusion or exclusion of those guidelines? | The systematic review aimed to gather evidence of odds of any risks of each mode of birth compared with the other (e.g. risk of urinary incontinence). We did not gather clinical guidelines in terms of recommendations. At the time of our systematic review (2020), we did not find any reports or statements from the WHO or the Ministry of Health in the 4 participating countries that provide risk estimates for short- and long-term maternal and child outcomes of women who planned for a CS compared to women who planned for a vaginal delivery. |
| • Throughout the paper, it would be helpful to streamline the repetitive writing: for example, Lines 221-225 repeats information that is available in table 2; lines 299-304 repeats information as well. Please consider revising the paper to reduce the repetitions. | Thank you for this suggestion. We reduced repetitions. |
| • In lines 353-355, the DALI registration and DAT meet 20 out of 22 standards; please add more information about what two standards were missed and if they are addressable. | We add more information about the two missed standards in the results section (page 18). We have already explained in the discussion section why these standards are missing and how we plan to remedy them (page 21). |
| • As noted above, the clear dissemination of the tool in paper and digital app is to be commended. Can the authors share information about the method of dissemination or ways that the tool is being shared beyond a peer-reviewed publication? How are the authors planning to get the tool in the hands of women and birthing people? | The overall strategy to implement the DAT is described in our QUALI-DEC research protocol (reference # 16). Trained health care providers will launch the DAT in their hospital. They will encourage antenatal care providers to deliver the DAT booklet to eligible pregnant women. This will require a series of on-site meetings in all relevant facilities to inform and motivate providers and to obtain their formal commitment. In addition, a DAT application will be developed for smartphones and made available in the settings in which it is considered culturally appropriate and most acceptable and convenient for women. Posters will be displayed on the wall of the waiting room of antenatal care centers with the QR code to access the web/smartphone application |
| MINOR: |  |
| • As noted in the PLoS Global Public Health best practices section on, “Inclusivity in Global Health,” it would be helpful to have the authors share their approach to authorship. | Thank you for this suggestion. We included PLOS’s questionnaire in supporting information. |
| • The abstract starts with the statement, “Women’s fear and uncertainty about vaginal delivery and lack of empowerment in decision-making generate decision conflict and the overuse of cesarean sections (CS) in LMICs.” This framing suggests that it is individual birthing people’s fault that CS rates are high when there are other significant factors at play, including the health system, cultural norms, facility level characteristics. Please consider reframing the background in the abstract or providing more information in the full background section about women’s role or lack thereof in decision making. | We reframed the background to suggest that women’s fear about vaginal delivery is one of the main determinants of high CS rates in LMICs. |
| • While this is obviously an editorial decision, I found the acronyms of CS (caesarean section) and VD (vaginal delivery) to be halting through the reading process. I am not sure if the word count would be deeply affected, but it would be easier on readers to avoid the acronyms if possible. | We deleted the acronyms. |
| • Under the Methods section (line 119-121), the authors state that the women with previous caesarean, breech, or abnormal presentation, or those with elective caesarean are not the target audience; it would be useful to understand “why?” for readers who do not work in maternal and newborn health. | We explained why these women are not eligible for the DAT, page 5 of the revised manuscript. Women with previous caesarean section, breech or abnormal presentation, twin pregnancy, or any indication for elective caesarean section (pre-labour) are not the target of the DAT because they are at high-risk for caesarean delivery. |
| • In lines 131-140, the authors present the goals and populations of the individual interviews and focus group discussions. How did the authors determine the number of interviews to conduct and what were the indicators for achieving saturation? | Maximum variation sampling was used to achieve a diverse sample of participants, to make sure that the findings are a reasonable reflection of the views and experiences of local stakeholders. This method uses prespecified parameters to stratify the sample and encourages recruitment and sampling based on diversity. We detailed recruitment and sampling of women and providers in the revised manuscript, page 6 and 9 |
| • In lines 139-140, the authors discussion the analysis of recordings and interpretation of data. Later in Step 3, it is shared that recordings and analysis were conducted in local language and then summarized and cross compared. Were the interviews in Step 1 retained and analyzed in Vietnamese? If translation was completed here (step 1 or step 3) please specify at what stage translation to English was done and by whom. | Recordings and analysis were conducted in local language in participating countries, including Vietnamese in Step 1. As specified page 9, the translation to English was done at the stage if the report interpreting country-level analyses. |
| • Line 163: Any specific reason Chinese articles were not included? | This was a pragmatic decision based on feasibility and budgetary constraints. We acknowledge this is a limitation. |
| • Table 2: Please consider adding the goal of each round of discussion s if they are different across the countries because the timing is different between the countries. | We clarified the Table 2 to describe which interviews were development of the DAT and which were performed after for assessment of the DAT |
| • Lines 306-308, can the authors describe what is meant by “safety” as a concern for women, or identify what women mean by safer? | The women interviewed referred to the safety of the baby, in terms of health risks |
| • Please provide more information on data accessibility and restrictions. | We detailed data accessibility and restrictions page 9 of the revised manuscript. According to our data management plan, qualitative interview transcripts in original language have been made available for QUALI-DEC researchers only. The translation into English of the thematic analysis (country-level report) will be made available to the public at the end of the project. |

Reviewer #3:

| Reviewer’s comment | Authors’ answer |
| --- | --- |
| This is a research article on aimed at developing a decision analysis tool (DAT) to help women with a low-risk pregnancy make an informed choice about the mode of delivery and to evaluate it’s acceptability in 3 LMICs. The background, methods and strengths are well described. |  |
| It’s unfortunate that due to the COVID-19 pandemic, interviews could not be done individually in Argentina. This undermines the qualitative analysis since the population of that country may be culturally significantly different from the others evaluated. This needs to be acknowledged in the limitations. | We add this limitation in the discussion section page 21. |
| It is stated in the article that this tool may benefit women who request a planned cesarean section, particularly in the private sector, however, one of the limitations that this tool may have is that no private hospitals participated in the study. This should also mentioned as a limitation. | We add this limitation in the discussion section page 21. |
| I could not locate and download the application in the Apple Store since the app is currently not available in my country or region. This needs to be addressed. | The App is available in the countries which are participating in the QUALI-DEC project. The App will be accessible in the other countries at the end of the project. We are sorry for that. |
| On page 18, line 368, “heath care” should be corrected to “health care”. | Thank you. Correction was made |
